# Exclusive inhibition of IL-6 trans-signaling by soluble gp130^FlyR^Fc

**DOI:** 10.1016/j.cytox.2021.100058

**Published:** 2021-11-29

**Authors:** Anna F. Berg, Julia Ettich, Hendrik T. Weitz, Matthias Krusche, Doreen M. Floss, Jürgen Scheller, Jens M. Moll

**Affiliations:** Institute of Biochemistry and Molecular Biology II, Medical Faculty, Heinrich-Heine-University, 40225 Düsseldorf, Germany

**Keywords:** IL-6, IL-11, Trans-signaling, Soluble gp130, Sgp130, Cytokine

## Abstract

gp130 is the signal-transducing receptor for the Interleukin (IL)-6 type cytokines IL-6 and IL-11. To induce signaling, IL-6 forms a complex with IL-6 receptor (IL-6R) and IL-11 with IL-11 receptor (IL-11R). Membrane-bound IL-6R and IL-11R in complex with gp130 and the cytokine mediate classic-signaling, whereas trans-signaling needs soluble IL-6R and IL-11R variants. Interleukin (IL)-6 trans-signaling is of particular importance because it drives the development of autoimmune diseases, including rheumatoid arthritis and chronic inflammatory bowel diseases, whereas a role for IL-11 trans-signaling remains elusive. Soluble gp130 selectively inhibits trans-signaling of IL-6 whereas both, classic- and trans-signaling are abrogated by IL-6- and IL-6R-antibodies. Recently, we described an optimized sgp130 variant, which carries three amino acid substitutions T102Y/Q113F/N114L (sgp130^Fly^Fc) resulting in reduced inhibition of IL-11 trans-signaling by increasing the affinity of sgp130 for the site I of IL-6. Moreover, we described that the patient mutation R281Q in gp130 results in reduced IL-11 signaling. Here, we show that the combination of T102Y/Q113F/N114L and R281Q in the new variant sgp130^FlyR^Fc results in complete preservation of IL-11 mediated trans-signaling, whereas inhibition of IL-6 trans-signaling is maintained. Since sgp130Fc (olamkicept) has successfully completed a phase IIa trial in Crohn’s disease (CD) and ulcerative colitis, sgp130^FlyR^Fc might serve as second-generation therapeutic to diminish IL-11 *trans*-signaling cross-reactivity.

## Highlights

• A variety of sgp130Fc muteins was generated.
• Introduction of a gp130 SNP (R281Q) into sgp130Fc increases IL-6 specificity.
• The sgp130Fc variant sgp130^FlyR^ exclusively affects IL-6 trans-signaling.

## Introduction

Interleukin (IL-)6 is the name-giving cytokine of the IL-6 type cytokine family, which consists of nine cytokines. Only IL-6 and IL-11 signal via homodimeric complexes of gp130. Specificity is conveyed by the specific alpha receptors, IL-6 receptor (IL-6R), and IL-11R. Thereby, cells expressing membrane-bound IL-6R can signal via IL-6 in a process called IL-6 classic signaling and cells expressing membrane-bound IL-11R signal via IL-11 classic-signaling. Since the expression pattern of IL-6R and IL-11R are different, the physiological consequences of IL-6 and IL-11 are different. Whereas IL-6 controls inflammatory processes including acute phase response, infection, and autoimmunity but is also involved in tumor development and metabolism [1], IL-11 is mainly involved in regenerative processes [2]. Alpha receptor binding by IL-6 or IL-11 is followed by recruitment of the ubiquitously expressed signal-transducing co-receptor gp130 and initiation of downstream signaling cascades leading to activation of the Janus kinase/signal transducer and activator of transcription (JAK/STAT), protein kinase B (PKB or AKT) and mitogen-activated protein kinase (MAPK) pathways. Alternative activation pathways include trans-signaling and trans-activation. In trans-signaling, IL-6/IL-11 binds to soluble IL-6R/IL-11R with the subsequent complex formation and signal initiation with homodimeric gp130 receptors. These complexes can activate downstream signaling cascades in all cells expressing gp130 [3]. Increased or dysregulated IL-6 trans-signaling contributes to the development of many chronic inflammatory diseases including rheumatoid arthritis [4], [5], multiple sclerosis [6], inflammatory bowel disease [7], [8], [9], and cancer [10], [11], [12]. IL-11 trans-signaling was described in humans but a functional role was not defined yet [13]. In IL-6 trans-presentation, IL-6 binds to membrane-bound IL-6 receptor on a transmitter cell, which also engages a homodimer of gp130 which is expressed on a juxtacrine receiver cell. Some dendritic cells lack gp130 but express IL-6R and trans-presentation is needed for some CD8 T cell responses [14], [15]. For IL-11, trans-presentation was described in cell culture but not yet verified to occur in living organisms [13]. For therapy, targeting IL-6 but not IL-11 is of high interest for many diseases. To date, inhibition of IL-6 signaling antibodies directed against IL-6 and the IL-6R have been approved for e.g. rheumatoid arthritis. Blockade via IL-6 antibodies is, however, associated with many side effects including an increased risk of bacterial and viral infections [16], [17]. Mouse studies indicated that blockade of classic-signaling by IL-6/IL-6R antibodies is unfavorable [18] and should be avoided by second-generation therapeutics which selectively target trans-signaling. To date, only sgp130Fc (Olamkicept) has been described to selectively targeting IL-6 trans-signaling (and trans-activation). The sgp130Fc variant olamkicept successfully completed a phase IIa trial in Crohn’s disease (CD) and ulcerative colitis (UC) (EudraCT: 2016-000205-36) [19] with promising outcomes including complete remission in 22% of the UC and 14% of the CD patients and clinical response in 44% of the UC and 55% of the CD patients, respectively. Currently, an additional phase II trial in ulcerative colitis (NCT03235752) is ongoing. However, sgp130Fc inhibits IL-11 trans-signaling with similar potency as IL-6 trans-signaling [13]. Of note, the mode of inhibition is completely different for antibodies targeting IL-6 or IL-6R and sgp130Fc targeting the complex of IL-6 and sIL-6R or IL-11 and sIL-11R. IL-6 and IL-11 have three binding sites for their receptors. The site I recognize IL-6R and IL-11R, respectively, while sites II and III establish the contact to gp130. IL-6 or IL-6R antibodies target the site I interface formed between IL-6 and the IL-6R and affect classic, trans-signaling, and trans-activation. Like the transmembrane receptor gp130, the fusion protein sgp130Fc, which consists of all six extracellular domains of gp130 fused to an Fc-part of an IgG antibody, targets sites II and III and does not affect IL-6 classic-signaling, unless extremely high concentrations of sIL-6R are used that allow quantitative complexing of IL-6 with sIL-6R [20]. Whereas domain 1 interacts with site III of IL-6, the cytokine-binding module (CBM, domains 2 and 3) interact with IL-6 via site II [21]. Domains 4 to 6 are fibronectin type III (FNIII)-like domains and not directly involved in cytokine binding. However, they are needed for activation of signal transduction and for unknown reasons support binding of IL-6:sIL-6R complexes since shorter sgp130 variants lacking the FNIII domains exhibit reduced inhibitory potency [22]. The Fly mutations (T102Y/Q113F/N114L) are located in the Ig-like D1 domain and increase the affinity towards binding site III of IL-6 of gp130 to increase the inhibitory capacity of sgp130Fc towards IL-6:sIL-6R complexes [23]. Just recently, we found a decreased affinity for Fly towards site III of IL-11 and thereby reduced inhibitory capacity of sgp130Fc towards IL-11:sIL-11R complexes [24]. Only IL-27 of the IL-6 family also interacts with the Ig-like D1 domain of gp130. IL-27 interacts with D1 of gp130 because the IL-27 receptor WSX1 has no Ig-like D1 domain. Therefore, it is possible, that the Fly mutations also affect the binding of gp130 to IL-27. However, we have previously shown that sgp130Fc cannot inhibit IL-27 signaling [25], and hence it is likely that also sgp130^Fly^Fc cannot inhibitIL-27 signaling. Albeit at were high concentrations leukemia inhibitory factor (LIF) and oncostatin M (OSM) signaling are slightly affected [26], [27], these and all other IL-6 type cytokines including CNTF, OSM, and LIF interact with gp130 via CBM which binding site is not affected by the Fly mutations [21]. Recently SNP R281Q was described for gp130 in a patient presenting with abnormal head shape and craniosynostosis and retained deciduous teeth [28]. We have shown that the R281Q substitution in domain 3 of the gp130 trans-membrane receptor confers reduced IL-11-signaling, while signaling of other IL-6 family members remained intact [28]. Here we incorporated the R281Q SNP into sgp130Fc and demonstrated that is displays an increased selectivity for the inhibition of IL-6 trans-signaling compared to IL-11 trans-signaling. Since sgp130^Fly^Fc and the novel variant sgp130^R281Q^Fc have reduced inhibitory capacity for IL-11 trans-signaling but maintained inhibition of IL-6 trans-signaling, we combined Fly and R281Q mutations in the new variant sgp130^FlyR^Fc. This resulted in a highly IL-6 specific trans-signaling inhibitor completely lacking effects on IL-11 mediated trans-signaling.

## Materials and methods

### Molecular cloning

A cDNA for human IL-11 was synthesized (Biocat GmbH, Heidelberg Germany) and subcloned into pcDNA3.1 via standard PCR methods. For the generation of sgp130^R281Q^Fc and sgp130^FLYR^Fc site-directed mutagenesis using the following primers (sgp130R281Q rv (5́-3́):ctgcactgtgaagctggactgggtggaagcggtatcctc and sgp130R281Qfw (5́-3́): gaggataccgcttccacccagtccagcttcacagtgcag) was performed on expression plasmids encoding sgp130Fc or sgp130^FLY^Fc templates [24]. HIL-11 was subcloned via HindIII and NotI in pcDNA3.1 vector (Invitrogen) containing N-terminal signal peptide and a C-terminal Twin-Strep-Tag, thereby generating pcDNA3.1-HIL-11-TS for secreted expression. An analogous cloning strategy was used to generate an expression vector for HIL-6-TS.

### Cells and reagents

The generation of Ba/F3-gp130 cells has been described elsewhere [29]. Ba/F3 cell lines were grown in DMEM high glucose culture medium (GIBCO®, Life Technologies, Darmstadt, Germany) supplemented with 10% fetal bovine serum (GIBCO®, Life Technologies), 60 mg/l penicillin and 100 mg/l streptomycin (Genaxxon bioscience GmbH, Ulm, Germany) at 37 °C with 5% CO_2_. Proliferation of Ba/F3-gp130 cells was maintained in the presence of hyper-IL-6 (HIL-6), a fusion protein of IL-6 and sIL-6R, which mimics the IL-6 trans-signaling complex [30]. Expression and purification of human hyper-IL-6 and human IL-6 were performed as described previously [30]. HEK293T (ACC-635) cells were purchased from the Leibniz Institute DSMZ-German Collection of Microorganisms and Cell Culture (Braunschweig, Germany). The Expi-293F™ cells (ThermoFisher Scientific) were cultured in 30 ml Expi293™ expression medium without antibiotics until they reached a density of 3–5 × 10^6^ cells/ml in a 37 °C incubator with 8 % CO2 on an orbital shaker at 125 rpm. The Expi293™ cells were subcultured in a shaker flask until they reached a density of 3–5 × 10^6^ cells/ml, typically every 3–4 days. Antibodies directed against STAT3 phosphorylated at Tyr705 (clone D3A7) and STAT3 (clone 124H6) were obtained from Cell Signaling Technology (Frankfurt, Germany). Rabbit anti-human IgG Fc (#31423) and peroxidase-conjugated secondary mAbs (#31432, #31462) were obtained from Pierce (Thermo Fisher Scientific, Waltham, MA, USA). Antibodies directed against pERK (#4370) and ERK (#4695) were obtained from Cell Signaling. Recombinant sgp130Fc and IL-6 were produced and purified as described previously [31]. Rabbit anti-human IgG Fc HRP conjugate was obtained from Thermo Fisher Scientific (Waltham, MA, USA). Recombinant soluble sgp130^FLY^Fc and sIL-6R were obtained from CONARIS Research Institute AG (Kiel, Germany). An expression plasmid for IL-11(Δ11)His_6_ lacking the first 11 amino acids of IL-11, was kindly provided by Prof. Dr. Christoph Garbers (Otto-von-Guericke-University Magdeburg). IL-11 was expressed as a soluble protein in *E. coli or Expi293 cells* and purified via affinity chromatography. The sIL-11R was obtained from Bio-Techne (Wiesbaden, Germany).

### Transfection, transduction, and selection of cells

CHO-K1 cells were cultured in DMEM medium. For expression of recombinant proteins, 5x10^5^ CHO-K1 cells were transfected with Turbofect (Thermo Fisher Scientific, Waltham, MA, USA) and 5 µg plasmid DNA encoding sgp130Fc variants. At 5 h after transfection, the medium was exchanged to DMEM medium without a transfection reagent. For the generation of stable CHO-K1 cell lines, G418 was added to the medium 48 h after transfection. Single clones were selected via limiting dilution. Positive clones expressing Fc fusion proteins were identified by Western blotting using anti-human Fc antibodies. HEK293T cells were cultured in DMEM medium. For expression of recombinant proteins, 2x10^6^ cells were transfected with Turbofect and 5 µg plasmid DNA encoding sgp130Fc variants. Mammalian expression plasmids encoding sgp130^R281Q^Fc, sgp130^FlyR^Fc, HIL-6-TS, IL-11-TS and HIL-11-TS were transfected into Expi-293F™ cells using ExpiFectamine™. Reaching 4.5–5.5 × 10^6^ cells/ml, the cells were diluted to a final density of 3 × 10^6^ cells/ml in 30 ml Expi293™ expression medium for transfection. 30 μg of the plasmid expression vectors were used for transfection. Henceforth, the culture was harvested by centrifugation at 450*g* at 4 °C for 5 min, followed by centrifugation of the resulting supernatant at 4000*g* at 4 °C for 20 min.

### Proliferation assays

Ba/F3-gp130 cells were washed and 5,000 cells of each cell line were cultured for three days in a final volume of 100 µl in the presence of cytokines and inhibitors. The CellTiter-Blue® Reagent was used to determine cellular viability by recording the fluorescence (excitation 560 nm, emission 590 nm) using an Infinite M200 PRO plate reader (Tecan, Crailsheim, Germany) immediately after adding 20 µl of reagent per well (time point 0) and up to 120 min thereafter. Fluorescence values were normalized by subtraction of time point 0 values. All experiments were performed at least three times, and one representative experiment was selected.

### Stimulation of Ba/F3 cells assays and lysate preparation

10^6^ Ba/F3-gp130 cells/ml and variants thereof were washed and starved in serum-free medium for 5 h. Prior to stimulation, cytokines and inhibitors were preincubated at room temperature for 30 min. Subsequently, cells were stimulated with the indicated cytokines and inhibitor combinations for 30 min, harvested by centrifugation at 4 °C for 5 min at 1500*g*, frozen and lysed. Protein concentration of cell lysates was determined by the BCA Protein Assay (Pierce, Thermo Scientific). Analysis of STAT3 activation was performed by Western blotting of 25–75 μg of total protein from total cell lysates and subsequent detection steps using the anti-pSTAT3 (Tyr705) (1:1000), anti-STAT3 (1:2000), anti-pERK (1:1000) ad anti-ERK (1:2000) antibodies described above.

### Western blotting

Proteins were separated by SDS–PAGE and transferred to polyvinylidene difluoride (PVDF) or nitrocellulose membranes. Membranes were blocked and probed with the indicated primary antibodies. After washing, membranes were incubated with secondary peroxidase-conjugated antibodies (1:2.500 dilution) or fluorescence-labeled secondary antibodies (1:10.000 dilution). The Immobilon^TM^ Western Reagents (Millipore Corporation, Billerica, MA, USA) and the ChemoCam Imager (INTAS Science Imaging Instruments GmbH, Göttingen, Germany) or the Odyssey Fc Imaging System (LI-CORE Biosciences, Bad Homburg, Germany) were used for signal detection. Control STAT3 and ERK blots were produced on a separate membrane using the same samples.

### Expression and purification of recombinant proteins

sgp130Fc was produced in stably transfected CHO-K1 cells (see above) using a roller bottle system, sgp130^R281Q^Fc and sgp130^FLYR^Fc were produced in Expi293 cells. CHO-K1 culture supernatants were harvested and centrifuged at 1,000*g* and 4 °C for 30 min, followed by centrifugation of the resulting supernatant at 10,000*g* at 4 °C for 30 min. The supernatant of the second centrifugation step was filtered (bottle top filter, 0.45-μm pore diameter; Nalgene; Rochester, NY) and purified by affinity chromatography. Before chromatography, the pH values of the filtered cell culture supernatants were adjusted to 7.4. The supernatant was loaded on a protein-A column (HiTrap protein A HP; GE Healthcare) at a flow rate of 1 ml/min. The column was then washed with 30 column volumes of PBS. Proteins were eluted at pH 3.2–3.5 using a 50 mM citric acid buffer. Fractions of 1 ml were collected. Fractions containing the protein peak were pooled, and the pH was adjusted to pH 7 with 1 M Tris. HIL-11 and HIL-6 constructs containing a C-terminal Twin-Strep-Tag were purified using Strep-Tactin resin (IBA cat. #2-5025-001) according to the manufacturer’s instructions. Proteins were buffer exchanged to PBS using illustra NAP 25 (GE Healthcare Life Sciences, Munich, Germany) columns. Protein concentration was determined by measuring absorbance at 280 nm, and samples were flash-frozen in liquid nitrogen. Protein quality was assessed by SDS-PAGE and Coomassie staining.

### Surface plasmon resonance

For surface plasmon resonance experiments, a Biacore X100 instrument (GE Healthcare Life Sciences) was used. Analysis was performed in multi-cycle mode. Experiments were carried out at 25 °C in PBS pH 7.4, composed of 137 mM NaCl, 2.7 mM KCl, 12 mM HPO_4_^2−^ und H_2_PO^4−^, and 0.05% (v/v) surfactant P20 (GE Healthcare). sgp130Fc variants were captured on a Protein A chip at a level of ∼ 500 response units (RUs). HIL-6 or HIL-11 were injected at a flow rate of 30 μl/min at increasing concentrations (0.1953–50 nM) with a replicate of the 12.5 nM concentration. The association of sgp130Fc variants in each defined concentration was monitored in periods of 120 sec, and the dissociation was measured in periods of 400 or 500 sec. Final graphs were fitted using a 1:1 binding model.

## Results

### Molecular design, expression and purification of sgp130^R281Q^Fc and sgp130^FlyR^Fc

**Fig. 1:**
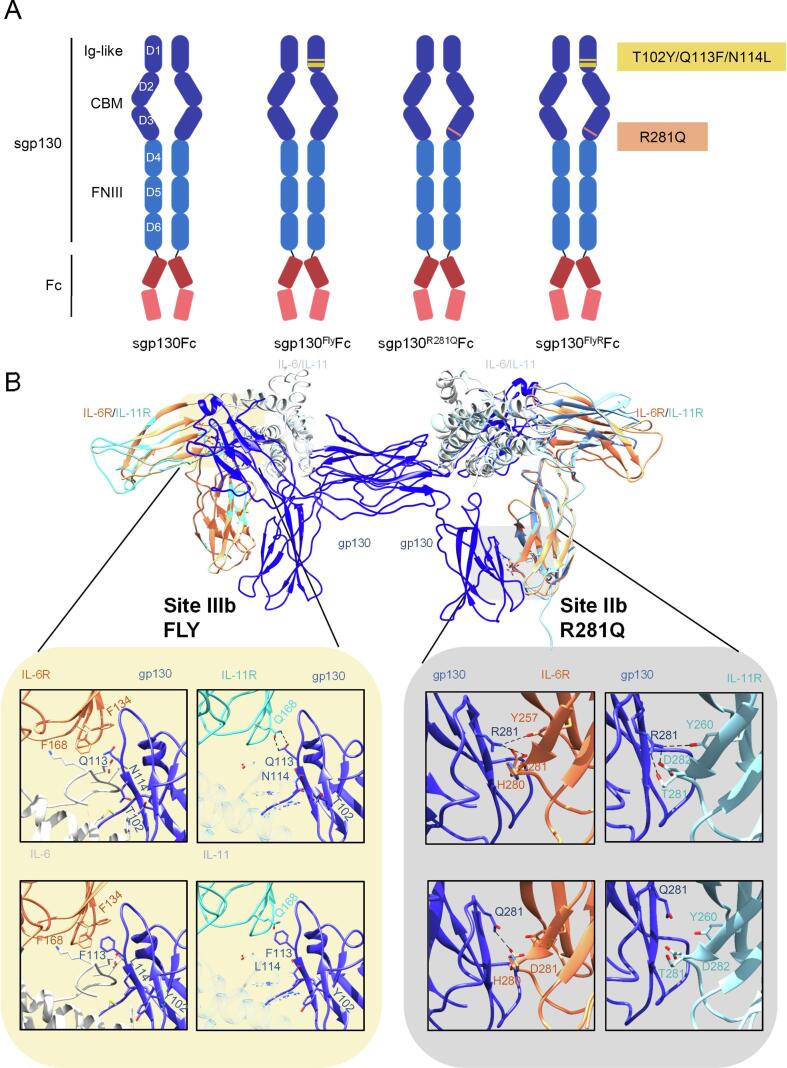
Design of sgp130^R281Q^Fc and sgp130^FlyR^Fc. (A) Protein domains are depicted as (not size-proportional) boxes. Domains D1-D3 (dark blue), domains D4-G6 (light blue) of gp130 were fused to the Fc fragment of a human IgG antibody (red). A TEV protease recognition sequence connects D6 to the Fc. Due to the presence of the Fc fragment, all depicted proteins are disulfide-linked dimers. Mutations in sgp130^R281Q^Fc, sgp130^Fly^Fc, and sgp130^FlyR^Fc are indicated in yellow and light red. (B) The crystal structure of the IL-6 signaling complex (PDB 1p9m) was superpositioned with the crystal structures of IL-11 (PDB 6o4o) and the IL-11R (PDB 6o4p) using UCFS Chimera. Contacts of gp130 formed at site IIb and III were then analyzed to evaluate the effects of FLY and R281Q mutation on the interaction of gp130 with the cytokine:α-receptor complexes. Electrostatic contacs are indicated by dotted lines. (For interpretation of the references to colour in this figure legend, the reader is referred to the web version of this article.)

cDNAs coding for sgp130Fc [27] and sgp130^Fly^Fc [24] were used as templates for site-directed mutagenesis to introduce the amino acid exchange R281Q (Fig. 1A). The Fly mutations are located in the Ig-like D1 domain and increase the affinity towards binding site III of IL-6. This may be due to the hydrophobic environment residues T102, Q113 and, N114 are located at site III (Fig. 1B), surrounded by F134, F168 (IL-6R), and L57 (IL-6). Mutation of those residues into Y102, F113, and L114, allows for more hydrophobic contacts e.g. between F113 of gp130 and F168 and F134 of IL-6R, thereby strengthening the binding interface. Superpositioning of IL-11R and IL-11 on the IL-6:IL-6R:gp130 structure (1p9m) indicates that IL-11 site IIIb is more electrostatic in nature. In this case e.g. Q113 of gp130 contacts Q168 of the IL-11R. As a consequence, the Fly mutations weaken the interaction with the IL-11R. Moreover, we have shown that the R281Q substitution in the membrane bound form of gp130 reduced IL-11-signaling, while signaling of other IL-6 family members remained intact [28]. The structural analysis demonstrated that R281 is located at the heart of the site IIb interface formed between gp130 and the IL-6R (Fig. 1B) [28]. There R281 of gp130 interacts via H-bonds and salt bridges with Y257 and D281 of the IL-6R or Y260, D282, and T281 of the IL-11R, respectively. Mutation of R281Q leads to a loss of the electrostatic interactions observed between gp130 and the IL-11R. Hence, R281Q mutation selectively weakens the interaction of gp130 and the IL-11R.

**Fig. 2:**
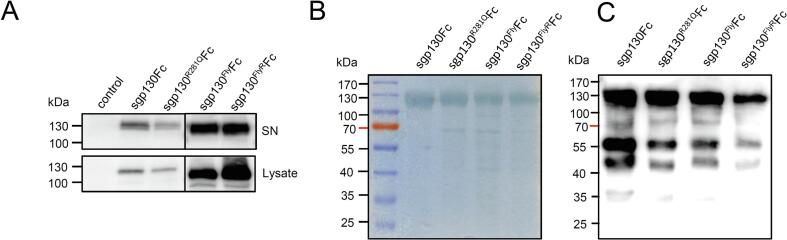
Expression and purification of sgp130^R281Q^Fc and sgp130^FlyR^Fc. (A) HEK293T cells were transfected with expression plasmids encoding the displayed proteins. Cells were harvested and lysed 48 h post-transfection. Supernatants and lysates were analyzed by sodium dodecyl sulfate–polyacrylamide gel electrophoresis (SDS-PAGE) and Western blotting using an antibody against human IgG-Fc. Western blots shown are representative of three different experiments with similar outcomes. (B + C) SDS-PAGE of purified sgp130Fc, sgp130^R281Q^Fc, sgp130^Fly^Fc, and sgp130^FlyR^Fc (5 μg of total protein) stained with Coomassie brilliant blue (B) and Western blotting (C) using an antibody anti-human IgG Fc HRP (500 ng of total protein). (For interpretation of the references to colour in this figure legend, the reader is referred to the web version of this article.)

cDNAs coding for sgp130^R281Q^Fc and sgp130^Fly,R281Q^Fc (sgp130^FlyR^Fc) were transiently transfected in Expi293 cells. Secretion of sgp130Fc, sgp130^Fly^Fc, sgp130^R281Q^Fc, and sgp130^FlyR^Fc of transiently transfected Expi293 cells was detected by Western blotting using Fc-antibodies (Fig. 2A). Following expression, proteins were purified via ProteinA sepharose chromatography (Fig. 2B and C). Proteins were pure based on SDS-PAGE analysis. In addition to full length proteins some degree of protein degradation was observed based on 55 and 45 kDa bands visible in Fig. 2C via Western blotting. Yields of 3.8 mg/L were obtained for sgp130Fc purified from 800 ml supernatants from stable CHO-K1 cells, while we obtained yields of approximately 13 mg/L for sgp130^Fly^Fc and sgp130^FlyR^Fc which were purified from 30 ml of transiently transfected Expi293 cells.

### sgp130^R281Q^Fc and sgp130^FlyR^Fc are potent inhibitors of IL-6 trans-signaling

**Fig. 3:**
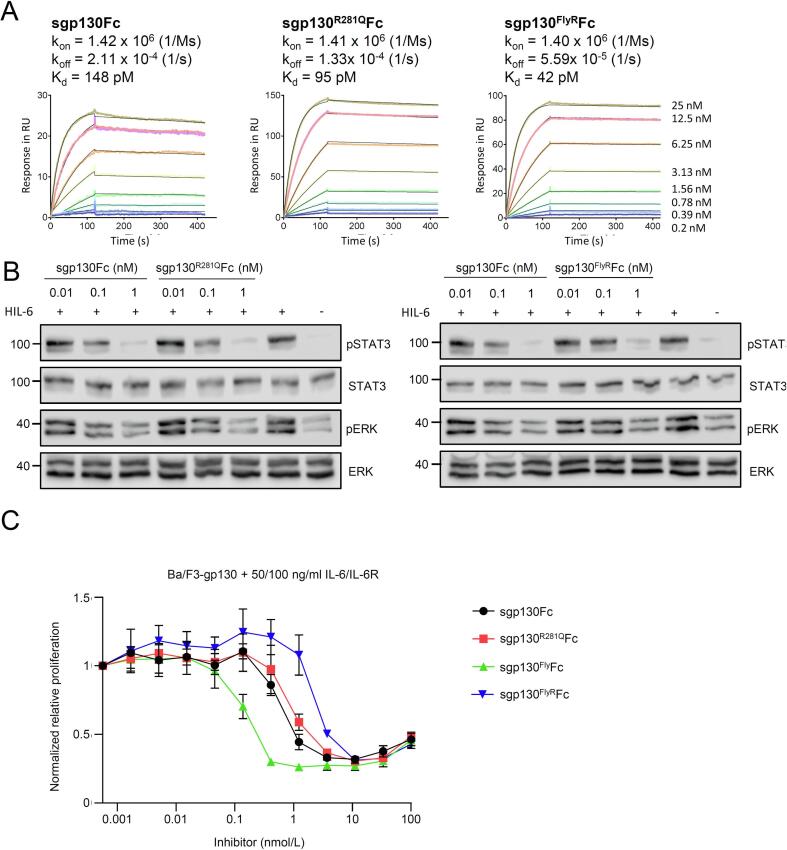
sgp130^R281Q^Fc and sgp130^FlyR^Fc are potent inhibitors of IL-6 trans-signaling. (A) Surface plasmon resonance analysis of HIL-6 binding to sgp130Fc, sgp130^R281Q^Fc, and sgp130^FlyR^Fc. sgp130Fc, sgp130^R281Q^Fc, and sgp130^FlyR^Fc were captured on a ProtA chip and increasing concentrations of HIL-6 were injected. Sensorgrams in response units (RU) over time are depicted as colored lines, global fit data are displayed as black lines. (B) Western blot analysis of Ba/F3-gp130 cells stimulated for 30 min with 10 ng/ml HIL-6 in the presence of the indicated concentrations of sgp130Fc, sgp130^R281Q^Fc, and sgp130^FlyR^Fc. Prior to stimulation, HIL-6 and inhibitors were incubated separately for 30 min. Western blots were stained for phosphorylated (p)STAT3, (p)ERK, STAT3 and ERK. Western blots are representative of three independent experiments. (C) Ba/F3-gp130 cells were stimulated with 50 ng/ml IL-6 and 100 ng/ml sIL-6R in the presence of increasing sgp130Fc, sgp130^R281Q^Fc, sgp130^Fly^Fc, and sgp130^FlyR^Fc concentrations. At 72 h post-stimulation, cellular proliferation was detected using a CellTiter-Blue assay. Normalization of relative proliferation was performed as described in Methods. Assays are representative of three independent experiments. (For interpretation of the references to colour in this figure legend, the reader is referred to the web version of this article.)

sgp130 is a potent inhibitor of IL-6 trans-signaling [27]. Introduction of the three amino acid exchanges T102Y/Q113F/N114L in sgp130 (sgp130^Fly^Fc) in binding site I of IL-6 increased affinity of sgp130 towards IL-6:sIL-6R complexes [23]. Using surface plasmon resonance, we compared the binding affinities of the established sgp130Fc, sgp130^Fly^Fc, and the novel variants sgp130^R281Q^Fc and sgp130^FlyR^Fc (Table 1). Binding affinities of sgp130Fc and sgp130^Fly^Fc to Hyper IL-6 (HIL-6) were determined previously [23], and are in good agreement with our control determination of sgp130Fc to HIL-6 of 148 pM. HIL-6 is a fusion protein of IL-6 and the soluble IL-6R connected by a flexible peptide linker. HIL-6 mimics trans-signaling and is a potent inducer of cells expressing gp130. Introduction of R281Q in sgp130^R281Q^Fc and sgp130^FlyR^Fc only minimally affects the binding affinity to HIL-6 compared to sgp130Fc (95 pM, 42 pM vs 148 pM, respectively) (Fig. 3A). This is supported by the capacity of sgp130^R281Q^ and sgp130^FlyR^Fc to inhibit HIL-6 induced STAT3 phosphorylation in Ba/F3-gp130 cells. The inhibitory kinetics of sgp130^R281Q^Fc and sgp130^FlyR^Fc are indistinguishable from sgp130Fc, about 1 nM sgp130Fc is sufficient for full inhibition of HIL-6 (0.2 nM) induced STAT3 phosphorylation, whereas 0.1 nM already resulted in slightly reduced STAT3 phosphorylation (Fig. 3B). Further support for the good inhibitory capacity of sgp130^R281Q^Fc and sgp130^FlyR^Fc were obtained from experiments using the inhibition of IL-6:sIL-6R-induced proliferation of Ba/F3-gp130 cells. Ba/F3-gp130 cells are commonly used in IL-6 cytokine research because proliferation depends on IL-6 trans-signaling. As shown previously, sgp130^Fly^Fc is a slightly more efficient inhibitor of trans-signaling induced cellular proliferation compared to sgp130Fc with IC50s of 0.27 nM and 0.45 nM, respectively. Importantly, sgp130^R281Q^Fc and sgp130^FlyR^Fc are also very potent inhibitors of trans-signaling induced cellular proliferation, with IC50s of 0.78 nM and 1.66 nM, respectively (Fig. 3C, Table 1). In conclusion, the established sgp130Fc and sgp130^Fly^Fc and the novel sgp130^R281Q^Fc and sgp130^FlyR^Fc variants are highly effective trans-signaling inhibitors with only minimal differences with regard to affinity and required inhibitory concentrations in cell-based assays.

**Table 1:** Affinities and inhibitory profiles of sgp130Fc variants for IL-6 and IL-11 trans-signaling complexes. Surface plasmon resonance analysis of hyper-IL-11-Fc binding to purified sgp130Fc and cs-130Fc. Hyper-IL-11-Fc was immobilized on a CM-5 chip and increasing concentrations of inhibitors were injected at a flow rate of 30 µl/min. Measurements were carried out on a a Biacore X100 instrument. Ba/F3-gp130 cells were stimulated with the indicated concentrations of cytokines and their respective soluble α-receptors in the presence of decreasing inhibitor concentrations. IC_50_ values were determined from three independent experiments.

Inhibitor	K_d_ (pM)	Stimulation cytokine (ng/ml)	IC_50_ (nM)
sgp130Fc	148 (HIL-6)	IL-6/sIL-6R (50/100)	0.45 ± 0.25
sgp130^Fly^Fc	122 (HIL-6 [23])	IL-6/sIL-6R (50/100)	0.18 ± 0.04
sgp130^R281Q^Fc	95 (HIL-6)	sIL-6R/IL-6 (50/100)	0.78 ± 0.40
sgp130^FlyR^Fc	42 (HIL-6)	sIL-6R/IL-6 (50/100)	1.66 ± 0.93
sgp130Fc		sIL-11R/IL-11 (10/100)	0.29 ± 0.16
sgp130^Fly^Fc		sIL-11R/IL-11 (10/100)	No inhibition
sgp130^R281Q^Fc		sIL-11R/IL-11 (10/100)	No inhibition
sgp130^FLY/R281Q^Fc		sIL-11R/IL-11 (10/100)	No inhibition
sgp130Fc	69 (HIL-11)	H-IL-11 (10)	0.12 ± 0.08
sgp130^Fly^Fc	670 (HIL-11)	H-IL-11 (10)	40.68 ± 30.43
sgp130^R281Q^Fc	610 (HIL-11)	H-IL-11 (10)	72.76 ± 39.80
sgp130^FLY/R281Q^Fc	N.A. (HIL-11)	H-IL-11 (10)	No inhibition

### sgp130^FlyR^Fc completely fails to inhibit IL-11 trans-signaling, whereas sgp130^Fly^Fc and sgp130^R281Q^Fc have reduced IL-11 trans-signaling inhibitory capacity

**Fig. 4:**
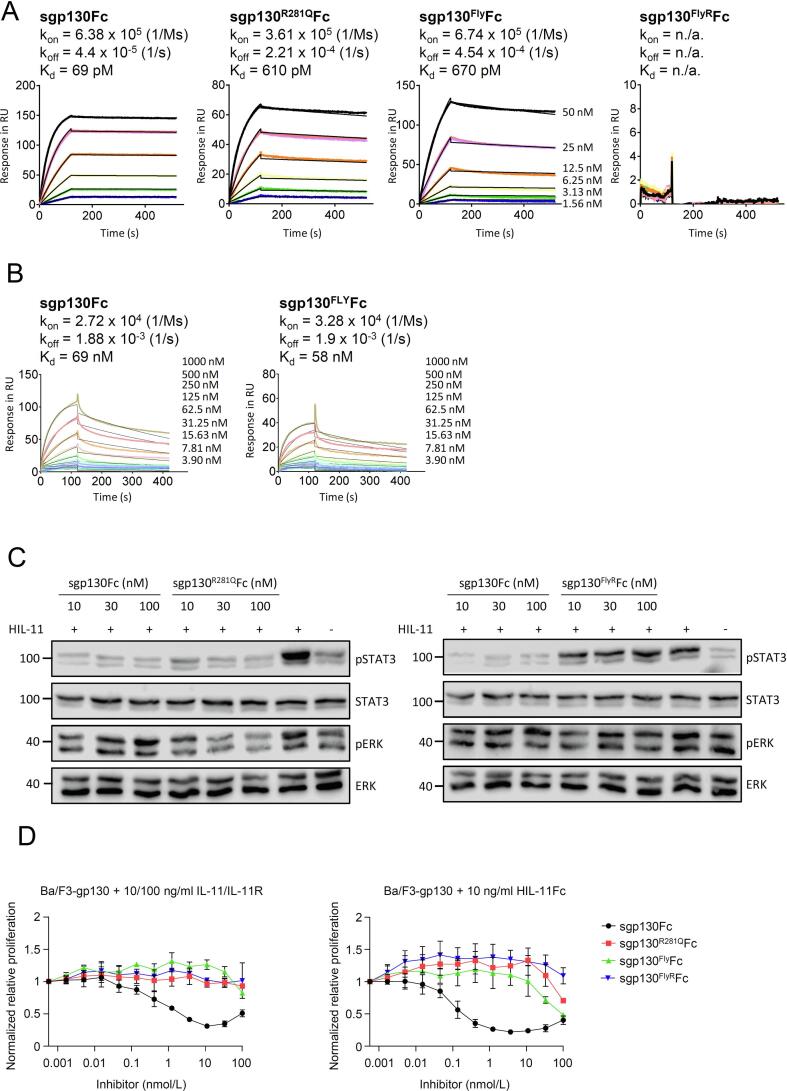
sgp130^FlyR^Fc completely fails to inhibit IL-11 trans-signaling, whereas sgp130^Fly^Fc and sgp130^R281Q^Fc have reduced IL-11 trans-signaling inhibitory capacity. (A) Surface plasmon resonance analysis of HIL-11 binding to sgp130Fc, sgp130^R281Q^Fc, sgp130^Fly^Fc, and sgp130^FlyR^Fc. sgp130Fc, sgp130^R281Q^Fc, sgp130^Fly^Fc and sgp130^FlyR^Fc were captured on a ProtA chip and increasing concentrations of HIL-11 were injected. Sensorgrams in response units (RU) over time are depicted as colored lines, global fit data are displayed as black lines. (B) Surface plasmon resonance analysis of IL-27 binding to sgp130Fc and sgp130^Fly^Fc. sgp130Fc and sgp130^Fly^Fc were captured on a ProtA chip and increasing concentrations of IL-27 were injected. Sensorgrams in response units (RU) over time are depicted as colored lines, fit data are displayed as black lines. (C) Western blot analysis of Ba/F3-gp130 cells stimulated for 30 min with 20 ng/ml HIL-11 in the presence of the indicated concentrations of sgp130Fc, sgp130^R281Q^Fc, and sgp130^FlyR^Fc. Prior to stimulation, HIL-11 and inhibitors were incubated separately for 30 min. Western blots were stained for phosphorylated (p)STAT3 and STAT3. Western blots are representative of three independent experiments. (D) Ba/F3-gp130 cells were stimulated with 10 ng/ml IL-11 and 100 ng/ml sIL-11R or 10 ng/ml HIL-11 in the presence of increasing sgp130Fc, sgp130^R281Q^Fc, sgp130^Fly^Fc and sgp130^FlyR^Fc concentrations. At 72 h post-stimulation, cellular proliferation was detected using a CellTiter-Blue assay. Normalization of relative proliferation was performed as described in Methods. Assays are representative of three independent experiments. (For interpretation of the references to colour in this figure legend, the reader is referred to the web version of this article.)

Like IL-6 in complex with sIL-6R, IL-11 in complex with sIL-11R also induces a homodimeric gp130 receptor signaling complex. Apparently, sgp130Fc is also a potent inhibitor of IL-11 trans-signaling [13]. Our previous results have shown that sgp130^Fly^Fc has reduced inhibitory capacity for IL-11 trans-signaling, which is likely a side product resulting from the introduction of affinity increasing amino acids for IL-6 binding [23], [24]. Here, we determined the affinities of sgp130Fc, sgp130^R281Q^Fc, sgp130^Fly^Fc, and sgp130^FlyR^Fc to HIL-11 by surface plasmon resonance (Table 1). Whereas the affinity of sgp130Fc to HIL-11 is highly comparable to HIL-6 (69 pM vs. 148 pM), sgp130^R281Q^Fc has an about 8fold reduced affinity towards HIL-11 (610 pM). Of note, we have observed a 6.4fold reduction in binding affinity of sgp130^R281Q^Fc for HIL-11 in comparison to HIL-6 (610 pM vs. 95 pM). A combination of these mutations in sgp130^FlyR^Fc, however, resulted in completely abrogated binding to HIL-11 as shown by surface plasmon resonance (Fig. 4A). Since the D1 domain of gp130 also interacts with IL-27, we further tested sgp130Fc and and sgp130^Fly^Fc for IL-27 binding via SPR (Fig. 4B). We found that both proteins bind to IL-27 with comparable affinities of 69 and 58 nM, respectively. Compared to their interaction with HIL-6 this is a reduction of affinity of approximately 1000 × fold. This is well in line with previously published results that sgp130Fc does not inhibit IL-27 signaling (25). In the latter study concentrations of 10 ng/ml sgp130Fc efficiently blocked HIL-6 induced IL-6 trans-signaling, while 1000 ng/ml sgp130Fc cannot inhibit IL-27 induced signal transduction. Based on our SPR data, concentrations of at least 10 μg/ml sgp130Fc or sgp130^Fly^Fc would be required to block IL-27 signaling. Whereas HIL-11 induced STAT3 phosphorylation in Ba/F3-gp130 cells was efficiently inhibited by sgp130Fc, the novel mutant sgp130^R281Q^Fc variant inhibited HIL-11 induced STAT3 phosphorylation with lower potency. 1 nM sgp130Fc was sufficient to completely block HIL-6 induced STAT phosphorylation (Fig. 3B), 10 nM of sgp130_R281Q_Fc were needed to obtain a reduction in H-IL-11 induced STAT3 phosphorylation (Fig. 4C). As expected from surface plasmon resonance experiments, even the highest inhibitor concentration of up to 100 nM sgp130^FlyR^Fc completely failed to inhibit HIL-11 induced STAT3 phosphorylation (Fig. 4C). Finally, the capacity to inhibit IL-11:sIL-11R and HIL-11 induced proliferation of Ba/F3-gp130 cells by sgp130Fc and variants thereof was determined. sgp130Fc inhibits IL-11:sIL-11R and HIL-11 induced cellular proliferation with IC50s of 0.29 nM and 0.12 nM, respectively (Fig. 4D). Inhibition of HIL-11 by sgp130Fc seemed to be more efficient, but only 0.1 nM (10 ng/ml) HIL-11 was used compared to 0.5 nM (10 ng/ml) IL-11 and 2.5 nM (100 ng/ml) sIL-11R. 5 fold fewer HIL-11 molecules compared to IL-11:sIL-11R molecules might explain, why less sgp130Fc was needed to inhibit HIL-11 compared to IL-11:sIL-11R induced proliferation. In addition, the nature of the hyper-cytokine may contribute to the better inhibition of HIL-11 compared to IL-11:sIL-11R complex. sgp130Fc only binds to the complex of IL-11:sIL-11R but not the individual components IL-11 or sIL-11R. During IL-11:sIL-11R treatment, only a fraction of cytokine and sIL-11R are complexed while probably the majority of the molecules is free. In contrast, due to the covalent linkage of IL-11 and sIL-11R in HIL-11, no diffusion of IL-11 and sIL-11R occurs. Hence, the equilibrium between free IL-11 and sIL-11R and the [IL-11:sIL-11R] complex is strongly shifted towards the complexed state. As a consequence, in comparison, more HIL-11 can be trapped by sgp130 compared to similar concentrations of IL-11 and sIL-11R. Since uncomplexed IL-11 and sIL-11R are not recognized by sgp130 variants, this may allow for free IL-11 and sIL-11R to form a complex in close proximity to membrane bound gp130. This may then lead to immediate recruitment of the complex by membrane bound gp130 resulting in signaling. Thus, the residual complex formation of IL-11 and sIL-11R close to membrane-bound gp130 may explain the reduced effects of sgp130 on IL-11:sIL-11R complexes. Therefore, only the highest concentration of sgp130^Fly^Fc (100 nM) was sufficient to at least slightly inhibit proliferation of IL-11:sIL-11R induced proliferation, whereas the highest concentration of sgp130^R281Q^Fc and sgp130^FlyR^Fc was not able to inhibit IL-11:sIL-11R induced proliferation. Hence, we used the more sensitive HIL-11 induced proliferation of Ba/F3-gp130 cells. Here we were able to reach sufficient concentrations of sgp130^Fly^Fc and sgp130^R281Q^Fc to demonstrate some inhibition HIL-11 induced cellular proliferation, with sgp130^R281Q^Fc being slightly less potent compared to sgp130^Fly^Fc (Fig. 3C). Noteworthy, the combination of these mutations in sgp130^FlyR^Fc failed to inhibit HIL-11 induced proliferation of Ba/F3-gp130 cells (Fig. 3C, Table 1). In conclusion, HIL-11 induced proliferation supports our findings from surface plasmon resonance and STAT3 phosphorylation demonstrating that the combination of R281Q with Fly in sgp130^FlyR^Fc resulted in the first complete IL-6 selective trans-signaling inhibitor based on the original sgp130Fc architecture.

## Discussion

In the present study, we describe the strictly IL-6 trans-signaling selective inhibitor sgp130^FlyR^Fc, which did not interfere with IL-11 trans-signaling. sgp130^FlyR^Fc was generated by introduction of the four amino acid exchanges T102Y/Q113F/N114L/R281Q into sgp130Fc. Several different endogenous soluble isoforms of gp130, implicated in the regulation of IL-6-type cytokine signal transduction, have been detected in human serum [22], [32], [33], [34]. Due to its implications in disease development, IL-6 is an important drug target and many therapeutic strategies were developed to inhibit excessive IL-6 signaling but only soluble gp130 variants were able to block trans-signaling specifically. We and others demonstrated that sgp130Fc is also a potent inhibitor of IL-11 trans- and cluster-signaling [13], [15], [35]. Therfore, the dissection of IL-6 and IL-11 trans-signaling effects by sgp130Fc is mandatory, because it cannot be excluded that phenotypes observed in sgp130Fc (mouse) models stem from combined effects on IL-6 and IL-11 signaling. IL-11 is a multifunctional cytokine with effects on hepatocytes, B-cells, macrophages, osteoclasts, cardiac myocytes and fibroblast hematopoietic cells including functions in cardiac regeneration and fibrosis, colon regeneration, bone metabolism and cancer [2], [36]. The first generation sgp130Fc (olamkicept) which blocks both IL-6 and IL-11 trans-signaling is currently developed as future therapeutic for chronic inflammatory bowel diseases. The sgp130Fc variant successfully completed a phase IIa trial in Crohn’s disease (CD) and ulcerative colitis (UC) [19]. Sgp130Fc induces minor adverse effects including upper respiratory infections, recurrence of herpes labialis and skin and subcutaneous disorders, suggesting that selective inhibition of trans-signaling by sgp130Fc is superior over combined inhibition of classic and trans-signaling by antagonistic IL-6 and IL-6R antibodies. Schreiber et al also recognized profound differences in the transcriptional signature in response to olamkicept and an antagonistic IL-6R antibody treatment, which were attributed to differences in the downstream effects of IL-6 classic and trans-signaling [19]. Effects on IL-11 trans-signaling might also be observed in this study, therefore, the described IL-6 trans-signaling selectivity of sgp130^FlyR^Fc may be the next important step towards second generation sgp130Fc variants. Due to its potential in blocking IL-6 trans-signaling, the first generation sgp130Fc variant was optimized in the past to improve stability and binding affinities for IL-6 [23]. Mutations at IL-6 binding sites II and III were described to improve binding affinities towards IL-6 resulting in improved biological activity [23]. Recently, we have shown that incorporation of the triple mutation FLY (T102Y/Q113F/N114L) at site III of sgp130Fc reduces inhibition of IL-11 trans-signaling, while the inhibitory activity towards IL-6 trans-signaling was maintained [24]. Moreover, we described that the patient mutation R281Q in gp130 also result in reduced IL-11 signaling [28]. Combination of Fly (T102Y/Q113F/N114L) and R281Q in sgp130^FlyR^Fc then resulted in the complete abrogation of IL-11 trans-signaling inhibition whereas inhibitory capacity towards IL-6 trans-signaling is maintained. In summary, sgp130^FlyR^Fc may be beneficial for the physiological and pathophysiological dissection of IL-6 family cytokine signaling modes. Furthermore, there are possible therapeutic applications of sgp130^FlyR^Fc as second generation IL-6 trans-signaling inhibitor.

## Declaration of Competing Interest

The authors declare the following financial interests/personal relationships which may be considered as potential competing interests: Juergen Scheller reports financial support was provided by German Research Foundation. Juergen Scheller, Jens M. Moll and Julia Ettich declare that they have a patent #EP-21189174-2 pending to HHU Düsseldorf.
